# Supplementary material for: The spread of the first introns in proto-eukaryotic paralogs
Source: Commun Biol. 2022 May 19;5:476. doi: 10.1038/s42003-022-03426-5 (PMC9120149; doi:10.1038/s42003-022-03426-5)
Supplement: Supplementary file 2 — Supplementary Information [file 42003_2022_3426_MOESM2_ESM.pdf]

Supplementary Information for:

The spread of the first introns in proto-eukaryotic paralogs

Julian Vosseberg, Michelle Schinkel, Sjoerd Gremmen, Berend Snel

Table of contents

Supplementary Notes 3
Supplementary Figures 6
Supplementary Tables 13
Supplementary References 17

Supplementary Notes

Supplementary Note 1: Potential intron transfer between paralogs

A third explanation for the presence of a shared intron between paralogs – next to vertical inheritance from a preduplication intron and parallel insertion in the same position – is the transfer of an intron from the intron-containing paralog to the other intron-lacking paralog. This transfer is proposed to occur via homologous recombination, resulting in ectopic gene conversion[1,2]. Three likely cases have been described in literature: a transfer of an intron between three globin paralogs in the insect *Chironomus*[2], between two ABC transporter paralogs in the ascomycete *Aspergillus*[3] and between two metalloprotease paralogs in the ascomycete *Mycosphaerella*[4]. Although these examples demonstrate that intron transfer between paralogs is a plausible mechanism, its relative contribution to shared introns between paralogs remains elusive.

To assess the possible impact of intron transfers between proto-eukaryotic paralogs, we looked more closely at the KOG clusters for which only one LECA intron position was shared between only two paralogs. The rationale for these criteria was that we think it is unlikely for multiple introns to be transferred or for an intron to be transferred between more than two paralogs. 148 LECA introns fulfilled these criteria for the KOGs, corresponding to 3.9% of the total number of shared LECA introns. For the Pfam OGs, 294 LECA introns were intron transfer candidates (11% of the total number of shared LECA introns). Given that intron transfers between paralogs and parallel insertions could have accounted for only a small number of shared LECA introns, we infer that most shared introns likely represent paralogous introns.

Supplementary Note 2: Differences in the detection of shared introns between different groups

For the Pfam OGs we analysed both the fraction of duplications with shared LECA introns and the fraction of LECA introns shared with paralogs. Differences can arise due to the phylogenetic relationships between OGs, which is ignored in calculating the fraction of LECA introns, and the large impact of a few clades on both numbers. For example, two intron-rich OGs with many shared introns can dominate the fraction of shared LECA introns of a category, while only reflecting a single duplication. Multiple paralogs that all share introns (i.e. a large fraction of duplications with pre-duplication introns) yet also have many OG-specific introns results in a low fraction of shared introns. When comparing both approaches, most differences are quite subtle.

For function (Fig. 2a, Supplementary Figure 5a) two categories were notably different. Energy metabolism had a lower (but still relatively high) fraction of shared LECA introns in comparison with the fraction of duplications with introns, for which it was the top category. The fraction of duplications with introns was dominated by mitochondrial carrier proteins (PF00153), which had shared introns traced to its first duplication and many later duplications. Conversely, duplications in amino acid metabolic genes had a higher fraction of shared LECA introns. This number was largely influenced by aminotransferases class I and II (PF00155) and serine hydroxymethyltransferase (PF00464) with 6 and 4 shared introns, respectively. Most of the OGs with this function in other Pfams had few, if any, LECA introns (Supplementary Figure 5b).

Whereas differences in duplication fractions between most cellular localisations were rather subtle, shared intron fractions revealed a more variable picture (Supplementary

Figure 5c). For the extracellular region most LECA introns were shared, in contrast with a relatively low fraction of duplications with introns. Ten duplications in leucine-rich repeat 8 (PF13855), which all shared the same three LECA introns, contributed to a large extent to this number, especially since OGs with this localisation had fewer introns in general (Supplementary Figure 5d). Introns could be traced back to the majority of duplications related to the endosome, whereas a lower fraction of LECA introns were shared between endosomal paralogs. Most of these duplications were in the PX domain (PF00787) and FYVE zinc finger domain (PF01363), with relatively few shared introns. Paralogs that function in the nuclear envelope did not share any LECA introns, which is in sharp contrast with 36% of nuclear envelope duplications sharing introns. Although these introns were shared between nuclear envelope and another (cytosol) and unknown localisation, the corresponding duplications in the RanBP1 (PF00638) and Importin-beta N-terminal domain (PF03810) had been annotated as duplications in nuclear envelope genes.

The most notable difference for the separate phylogenetic origins was the higher fraction of shared introns for alphaproteobacterial-related paralogs and the lower fraction of shared introns for Asgard archaea-related paralogs (Supplementary Figure 5e). OGs from probable endosymbiont origin had few introns in general, whereas OGs that were probably inherited from the host had more introns (Supplementary Figure 5f). The low number of LECA introns in alphaproteobacteria-related OGs could account for the low fraction of duplications with introns in alphaproteobacterial acquisitions.

Supplementary Note 3: The emergence of two different intron types

Two types of spliceosomal introns emerged during eukaryogenesis: U2 and U12. Three different models for the appearance of two types have been proposed[5].The first is the codivergence model, which postulates that the snRNA genes and introns diverged into two different sets after duplication of the snRNA genes. According to the fission/fusion model the two intron types evolved in separate proto-eukaryotic lineages that later fused. Whereas in the first two models the two types originated from primordial spliceosomal introns, in the parasitic invasion model the two types represent two temporally separate invasions of self-splicing group II introns.

To investigate the origin of the two different types of introns during eukaryogenesis, we predicted the type of all introns. 1.5% (KOGs) and 1.9% (Pfams) of LECA introns were predicted to be of U12-type, which was higher than in any present-day eukaryote in our dataset. 14.8% (KOGs) and 31.1% (Pfams) of these U12-type LECA introns were shared with an intron in a paralog, which is not significantly lower than for U2-type introns (Fisher's exact tests, P = 0.072 (KOGs) and P = 0.53 (Pfams)). Most of these shared U12-type LECA introns in KOGs were paired to U2-type introns in paralogs (56 U12-U2 pairs and 4 U12-U12 pairs). In contrast, 29 of the 50 shared U12-type LECA introns in Pfams were paired with at least one other U12-type LECA intron in a paralog. The higher numbers of U12-U12 pairs in the Pfams set may result from multiple *bona fide* OGs being combined into a single KOG (see main text).

U12-type introns were less often in phase 0 and more in phase 2 than U2-type introns (Supplementary Figure 7a, Supplementary Figure 8a) and even more biased towards the 5' end (Supplementary Figure 7b). Both observations are consistent with previous studies comparing intron types in present-day eukaryotes[6,7]. Assuming that nearly all type conversions were from U12 to U2 conversion, as has been argued based on comparative analyses[8], we inferred 32 U12 gains, 9 complete U12 losses and 7 U12-to-U2 conversions

before duplications and a further 113 U12 gains, 20 complete U12 losses and 15 U12-to-U2 conversions on the branches that resulted in the LECA families. Paralogs that had a U12-type intron traced back to their pre-duplication lineage were overrepresented in cell cycle and inorganic ion transport and metabolism functions (Supplementary Figure 8b). Differences in the fraction of U12-type introns among shared introns between functions of KOGs were not significant (Supplementary Figure 7c) and only a few significant differences between different phylogenetic origins of these paralogs were found (Supplementary Figure 8c). U12-type introns emerged at least before a large part of the complexification of the cell cycle and comparisons of the branch lengths seemed to suggest that U12-type introns are as old as U2-type introns, if not older (Supplementary Figure 6b).

The three models have different expectations regarding shared U12-type introns, depending on the timing of minor intron emergence. The occurrence of both types among shared introns and the inferred age of U12-type introns are not consistent with the model of two invasions by group II introns that were clearly separated in time. Divergence from primordial introns in either separate lineages followed by fusion of these lineages or divergence in the same lineage is a more likely scenario based on our findings.

Supplementary Figures

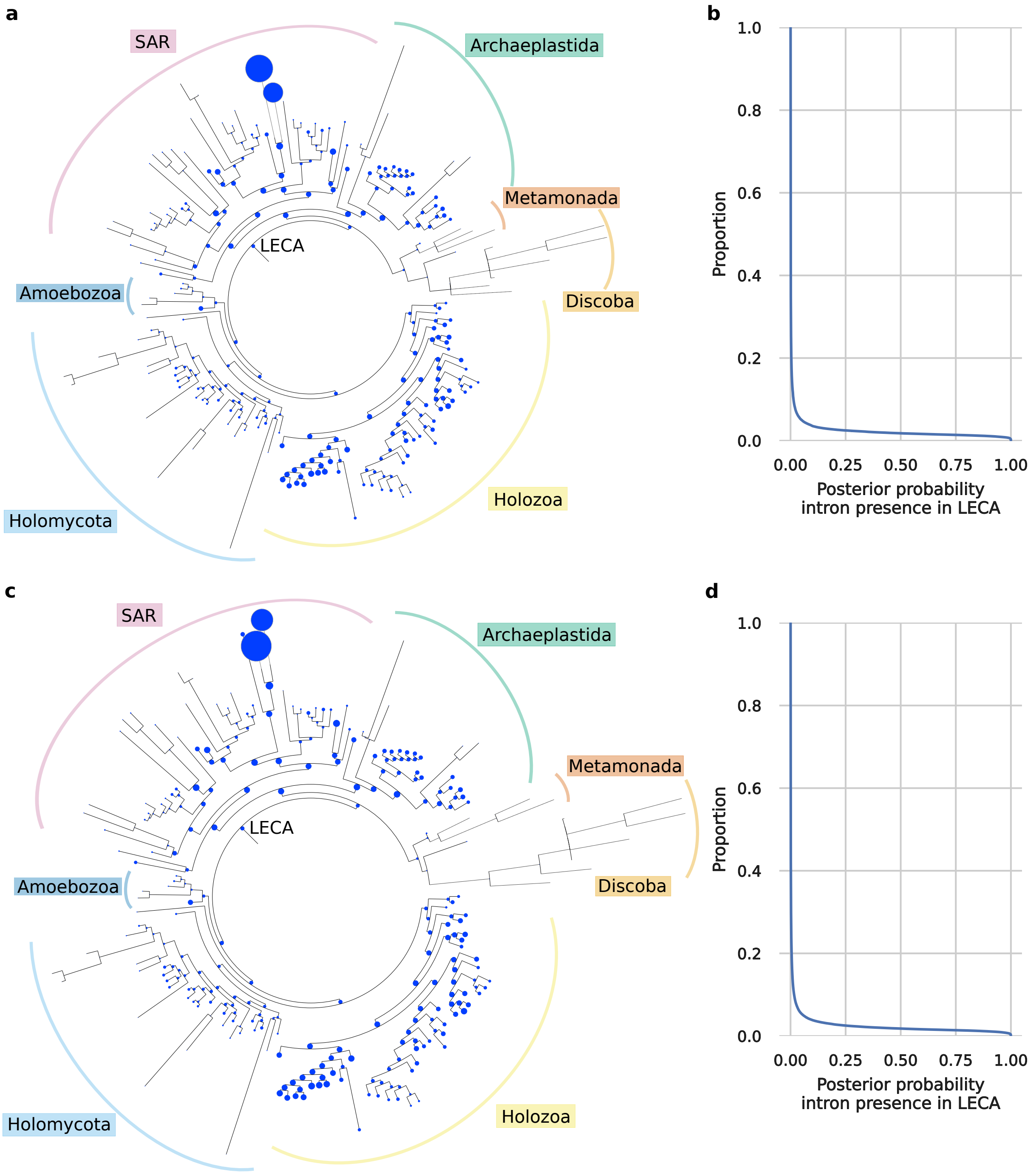

Supplementary Figure 1. Ancestral intron reconstructions. a Species tree with for each node the estimated number of introns (including missing sites) in KOGs represented as circles. These estimates are based on all used KOGs, including those from separate acquisitions. The size of the LECA node corresponds to 27,108 introns. The two terminal nodes with the highest number of introns correspond to two dinoflagellate species. **b** Descending empirical cumulative distribution function plot of the posterior probability of a KOG intron to have been present in LECA. **c** Species tree with for each node the estimated number of introns (including missing sites) in Pfam OGs represented as circles. These estimates are based on all used Pfam OGs, including those from separate acquisitions. The size of the LECA node corresponds to 14,977 introns. **d** Descending empirical cumulative distribution function plot of the posterior probability of a Pfam OG intron to have been present in LECA.

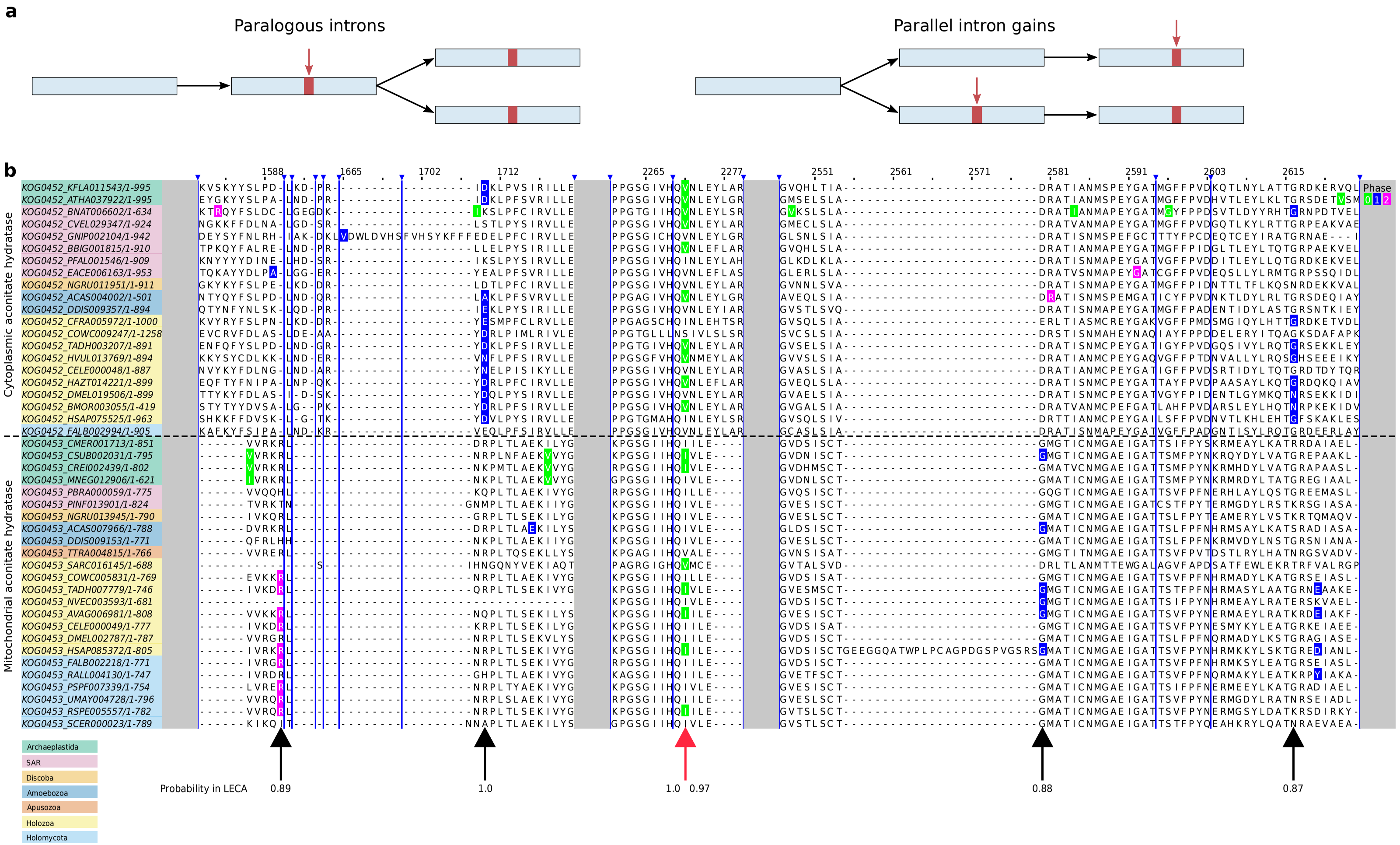

Supplementary Figure 2. Parallel intron gains. a Intron positions that are shared between paralogs could represent paralogous introns or could be due to parallel intron gains. **b** Example of parallel intron gains in separate acquisitions. Several aligned sequences from KOG0452 (cytoplasmic aconitate hydratase) and KOG0453 (mitochondrial aconitate hydratase) are shown with their mapped introns. The phase of introns is indicated with colours. Arrows point to introns that were probably present in LECA, with the number corresponding to the posterior probability. The shared intron that has been the result of parallel intron gains is indicated with a red arrow. Different sections of the alignment are separated by grey blocks and the blue stripes correspond to blocks of alignment positions that are only gaps in the sequences shown.

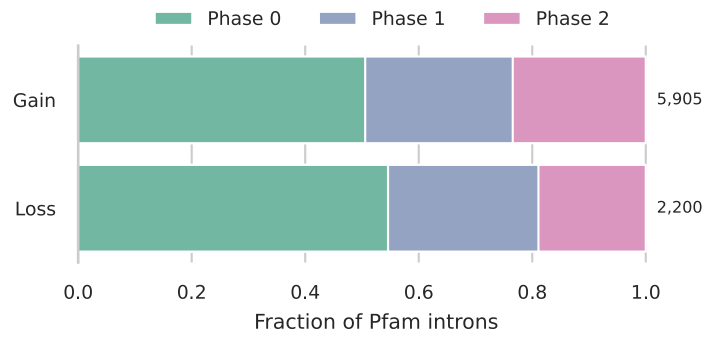

Supplementary Figure 3. Phase distributions of introns in Pfams that were gained or lost before LECA. Numbers indicate the number of inferred intron gains and losses.

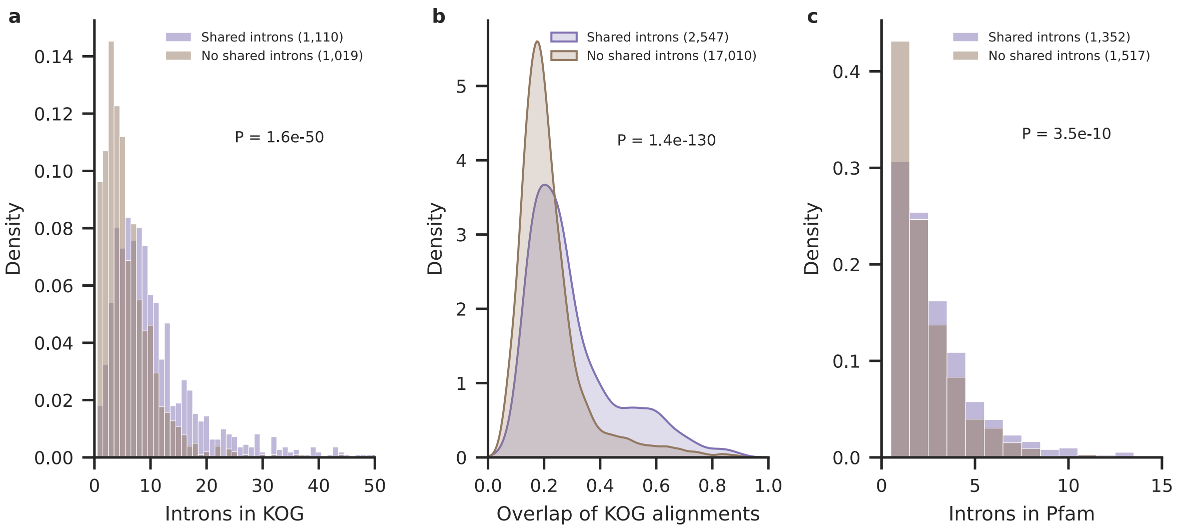

Supplementary Figure 4. Influence of number of LECA introns and overlap of OG alignments on finding shared introns. a Normalised histograms showing the distribution of the number of LECA introns in a KOG for KOGs with and without shared introns. For clarity, KOGs with more than 50 LECA introns are not shown. **b** Density plots showing the distribution of the fraction of overlapping positions in the alignment of all pairs of KOGs in the same cluster. Pairs with and without shared introns are depicted separately. Sites with more than 90% gaps were excluded in calculating the overlapping fraction. A lower overlap could be due to domain accretion and loss after duplication. **c** Normalised histograms showing the distribution of the number of LECA introns in a Pfam OG for Pfam OGs with and without shared introns. For clarity, Pfam OGs with more than 15 LECA introns are not shown. *P* values of Kolmogorov-Smirnov tests are shown. The numbers indicate the number of OGs (**a**, **c**) or pairs of KOGs (**b**). KOGs and Pfam OGs with no LECA introns were not included.

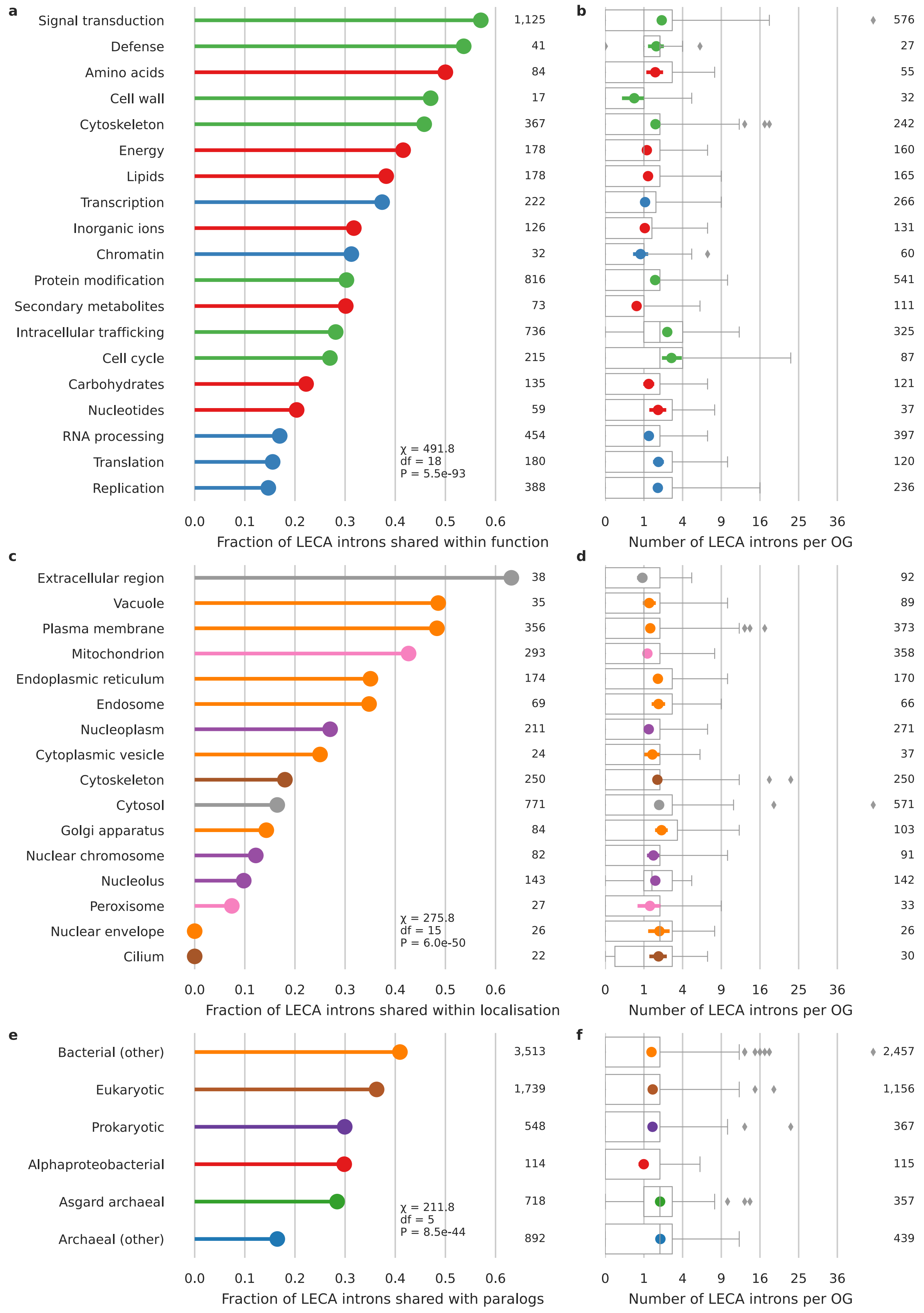

Supplementary Figure 5. Fraction of shared LECA introns and number of LECA introns for Pfam OGs. a, c Fraction of LECA introns shared between pairs of Pfam OGs with the same function (**a**) or localisation (**c**). Only functions and localisations with at least ten LECA introns and ten pairs are shown. Comparisons of the three functional categories were all significant (Supplementary Table 7), as were 50% of pairwise

comparisons (Supplementary Data 6). 60% of the comparisons between the five localisation categories and 58% of pairwise comparisons were significant (Supplementary Table 8, Supplementary Data 7). **e** Fraction of LECA introns shared between Pfam OGs within an acquisition or invention clade. 73% of pairwise comparisons were significant (Supplementary Table 9). **b**, **d**, **f** Number of LECA introns in an OG with a certain function (**b**), localisation (**d**) or phylogenetic origin (**f**) on a square-root scale. Distributions are shown with boxplots and the average number of introns per group with a coloured dot. Coloured bars represent 95% confidence intervals of the mean. Numbers correspond with the number of LECA introns (**a**, **c**, **e**) or number of Pfam OGs (**b**, **d**, **f**).

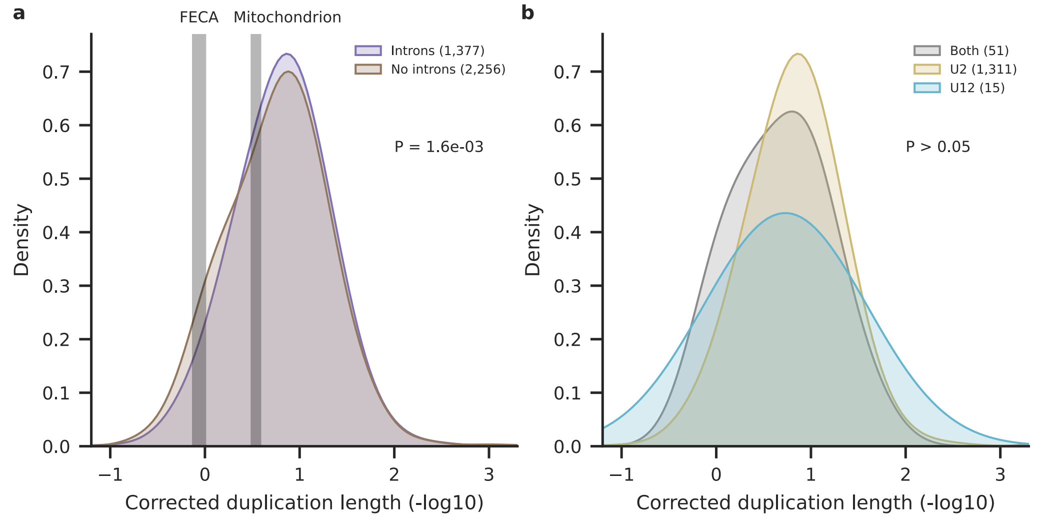

Supplementary Figure 6. Timing of duplications with introns using branch lengths from phylogenetic trees. a Density plot showing the duplication lengths of duplications with and without pre-duplication introns. For comparative purposes, the estimated timing[9] of the first eukaryotic common ancestor ('FECA'), which represents the divergence from the Asgard archaeal lineage, and the divergence from the alphaproteobacterial lineage ('Mitochondrion') are depicted in grey. The two distributions are significantly different according to the Kolmogorov-Smirnov test. **b** Density plot showing the duplication lengths of duplications with only U2-type, only U12-type or both types of pre-duplication introns. All pairwise comparisons with Kolmogorov-Smirnov tests were not significant. Numbers in both panels indicate the number of duplications considered.

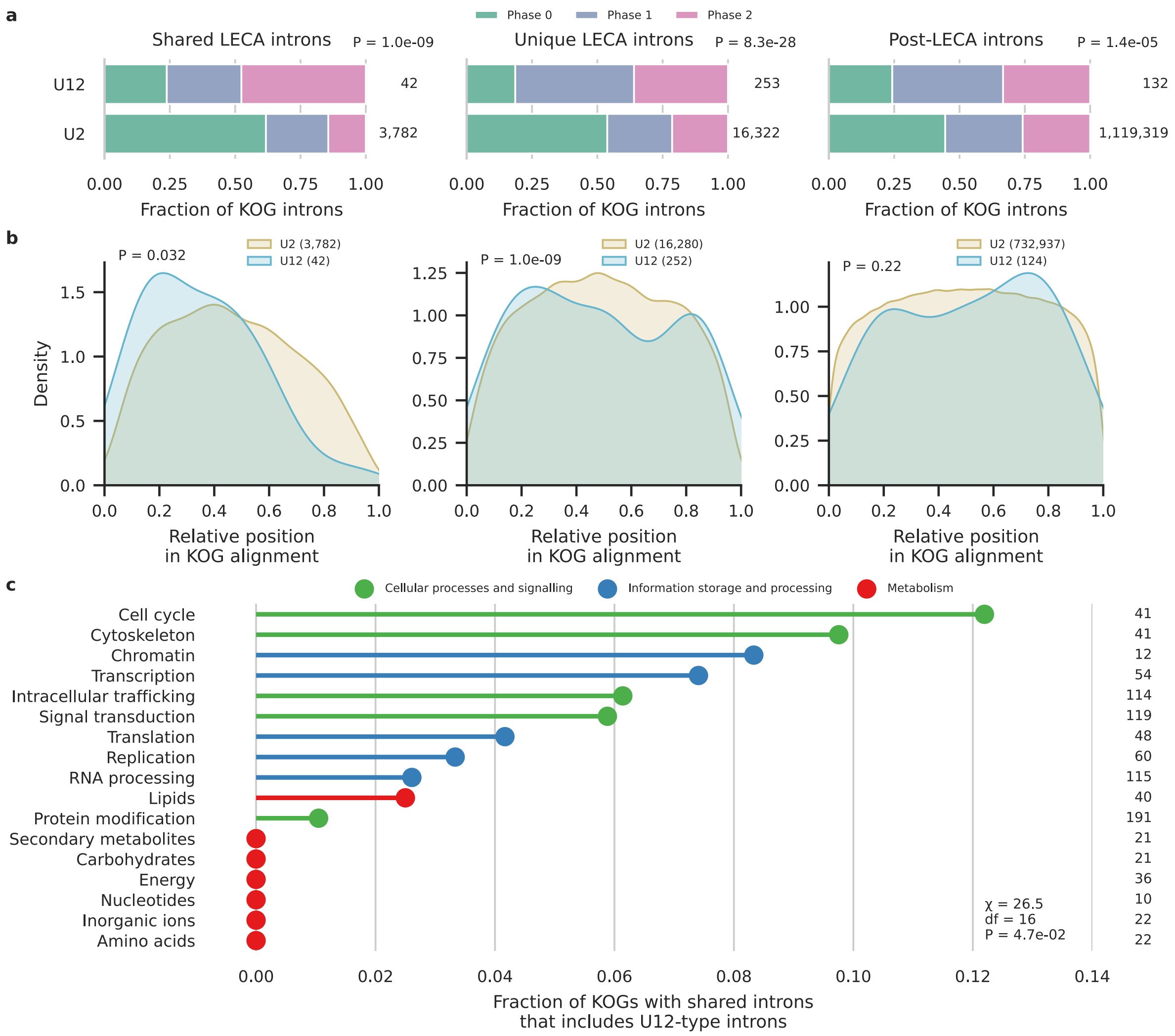

Supplementary Figure 7. Comparison of U2- and U12-type introns in KOGs. a Intron phase distributions for U2- and U12-type post-LECA, unique LECA and shared LECA introns in KOGs. *P* values of χ^2 contingency tests are shown. **b** Density plots showing the relative positions of introns in the alignment of a KOG, comparing U2- and U12-type introns for the three different groups. *P* values of Kolmogorov-Smirnov tests are shown. **c** Fraction of KOGs with shared introns that includes U12-type introns in the different functional categories. Only functions with at least ten KOGs with shared introns are shown. Comparisons of the three functional categories (Supplementary Table 10) and pairwise comparisons of the different functions (Supplementary Data 8) were not significant. Numbers indicate the number of introns considered (**a**, **b**) or the number of KOGs with shared introns (**c**).

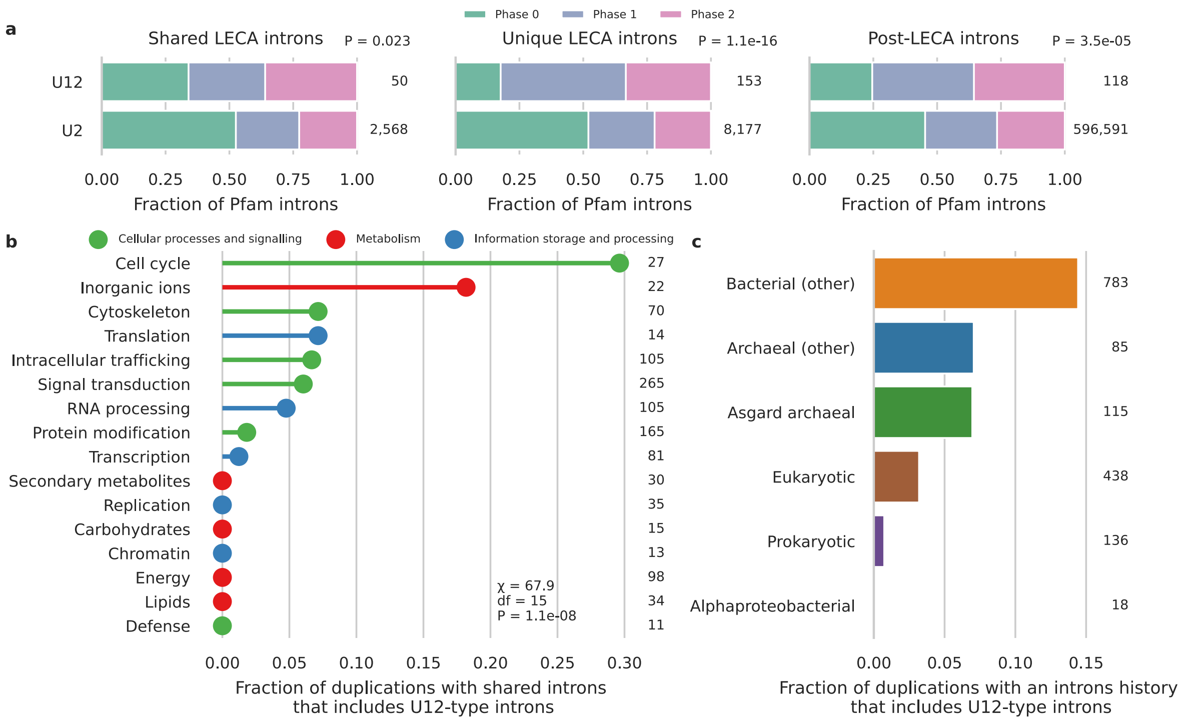

Supplementary Figure 8. Comparison of U2- and U12-type introns in Pfams. a Intron phase distributions for U2- and U12-type post-LECA, unique LECA and shared LECA introns in Pfam OGs. *P* values of χ^2 contingency tests are shown. **b** Fraction of Pfam duplications with pre-duplication introns that includes U12-type introns in the different functional categories. Only functions with at least ten duplications with shared introns are shown. Comparisons of the three functional categories were not significant (Supplementary Table 11). 9.2% of pairwise comparisons were significant, which were only comparisons including the cell cycle and inorganic ions (Supplementary Data 9). **c** Fraction of Pfam duplications with pre-duplication introns in either that duplication or a more ancestral duplication that includes U12-type introns according to the different phylogenetic origins. Only the pairwise comparisons of bacterial and eukaryotic and bacterial and prokaryotic duplications were significant (Supplementary Table 12). Numbers indicate the number of introns (**a**) or the number of duplications (**b**, **c**) considered.

Supplementary Tables

Supplementary Table 1. Pairwise comparisons of intron phases in KOGs.

Introns type 1	Introns type 2	χ^2	df	*P* value	Adjusted *P* value
Unique LECA introns	Shared LECA introns	111	2	6.61E-25	6.61E-25
Unique LECA introns	Post-LECA introns	506	2	1.46E-110	4.37E-110
Shared LECA introns	Post-LECA introns	467	2	3.66E-102	5.49E-102

Supplementary Table 2. Pairwise comparisons of intron phases in Pfams.

Introns type 1	Introns type 2	χ^2	df	*P* value	Adjusted *P* value
Unique LECA introns	Shared LECA introns	2.22	2	0.329	0.329
Unique LECA introns	Post-LECA introns	133	2	1.05E-29	3.15E-29
Shared LECA introns	Post-LECA introns	50.1	2	1.32E-11	1.98E-11

Supplementary Table 3. Pairwise comparisons of functional categories of pairs of KOGs with and without introns (unique versus shared introns).

Category 1	Category 2	Odds ratio	*P* value	Adjusted *P* value
Cellular processes and signalling	Metabolism	1.60	1.62E-15	2.44E-15
Cellular processes and signalling	Information storage and processing	2.74	3.35E-78	1.00E-77
Metabolism	Information storage and processing	1.72	4.29E-13	4.29E-13

Supplementary Table 4. Pairwise comparisons of functional categories of Pfam duplications with and without introns (duplications with versus without preduplication introns).

Category 1	Category 2	Odds ratio	*P* value	Adjusted *P* value
Cellular processes and signalling	Metabolism	1.44	4.89E-4	7.33E-4
Cellular processes and signalling	Information storage and processing	1.87	2.30E-11	6.89E-11
Metabolism	Information storage and processing	1.30	0.0249	0.0249

Supplementary Table 5. Pairwise comparisons of localisation categories of Pfam duplications with and without introns (duplications with versus without preduplication introns).

Category 1	Category 2	Odds ratio	*P* value	Adjusted *P* value
Endomembrane system	Metabolic compartment	1.18	0.334	0.418
Endomembrane system	Other	1.19	0.260	0.371
Endomembrane system	Nucleus	1.59	0.00651	0.0651
Endomembrane system	Cytoskeleton	1.69	0.0168	0.0841
Metabolic compartment	Other	1.01	1.00	1.00
Metabolic compartment	Nucleus	1.36	0.107	0.206
Metabolic compartment	Cytoskeleton	1.44	0.123	0.206
Other	Nucleus	1.34	0.0917	0.206
Other	Cytoskeleton	1.42	0.113	0.206
Nucleus	Cytoskeleton	1.06	0.821	0.912

Supplementary Table 6. Comparison of phylogenetic origins of Pfam duplications with and without introns (duplications with versus without preduplication introns).

Phylogenetic origin 1	Phylogenetic origin 2	Odds ratio	*P* value	Adjusted *P* value
Eukaryotic	Prokaryotic	1.09	0.532	0.570
Eukaryotic	Asgard archaeal	1.17	0.281	0.338
Eukaryotic	Bacterial (other)	1.40	7.97E-05	2.69E-04
Eukaryotic	Archaeal (other)	2.35	1.09E-09	1.64E-08
Eukaryotic	Alphaproteobacterial	3.46	1.26E-05	6.95E-05
Prokaryotic	Asgard archaeal	1.07	0.725	0.725
Prokaryotic	Bacterial (other)	1.28	0.0630	0.0946
Prokaryotic	Archaeal (other)	2.15	1.39E-05	6.95E-05
Prokaryotic	Alphaproteobacterial	3.16	1.47E-04	3.15E-04
Asgard archaeal	Bacterial (other)	1.19	0.213	0.291
Asgard archaeal	Archaeal (other)	2.01	1.16E-04	2.89E-04
Asgard archaeal	Alphaproteobacterial	2.95	5.26E-04	9.85E-04
Bacterial (other)	Archaeal (other)	1.69	8.95E-05	2.69E-04
Bacterial (other)	Alphaproteobacterial	2.48	0.00181	0.00302
Archaeal (other)	Alphaproteobacterial	1.47	0.293	0.338

Supplementary Table 7. Pairwise comparisons of functional categories of pairs of Pfam OGs with and without introns (unique versus shared introns).

Category 1	Category 2	Odds ratio	*P* value	Adjusted *P* value
Cellular processes and signalling	Metabolism	1.30	0.00115	0.00115
Cellular processes and signalling	Information storage and processing	2.75	3.34E-42	1.00E-41
Metabolism	Information storage and processing	2.12	1.17E-13	1.76E-13

Supplementary Table 8. Pairwise comparisons of localisation categories of pairs of Pfam OGs with and without introns (unique versus shared introns).

Category 1	Category 2	Odds ratio	*P* value	Adjusted *P* value
Metabolic compartment	Endomembrane system	1.07	0.632	0.703
Metabolic compartment	Other	2.87	8.85E-13	3.64E-12
Metabolic compartment	Nucleus	2.88	1.70E-10	3.40E-10
Metabolic compartment	Cytoskeleton	3.32	4.57E-10	7.61E-10
Endomembrane system	Other	2.67	9.75E-18	9.75E-17
Endomembrane system	Nucleus	2.69	1.09E-12	3.64E-12
Endomembrane system	Cytoskeleton	3.09	2.06E-11	5.15E-11
Other	Nucleus	1.01	1.00	1.00
Other	Cytoskeleton	1.16	0.467	0.668
Nucleus	Cytoskeleton	1.15	0.545	0.681

Supplementary Table 9. Comparison of phylogenetic origins of shared introns in Pfam OGs (unique versus shared introns).

Phylogenetic origin 1	Phylogenetic origin 2	Odds ratio	*P* value	Adjusted *P* value
Bacterial (other)	Eukaryotic	1.22	0.00119	0.00199
Bacterial (other)	Prokaryotic	1.62	7.56E-07	1.89E-06
Bacterial (other)	Alphaproteobacterial	1.63	0.0197	0.0269
Bacterial (other)	Asgard archaeal	1.75	1.97E-10	9.86E-10
Bacterial (other)	Archaeal (other)	3.51	6.32E-46	9.47E-45
Eukaryotic	Prokaryotic	1.33	0.00641	0.00962
Eukaryotic	Alphaproteobacterial	1.34	0.190	0.238
Eukaryotic	Asgard archaeal	1.43	1.77E-04	3.80E-04
Eukaryotic	Archaeal (other)	2.89	2.38E-27	1.79E-26
Prokaryotic	Alphaproteobacterial	1.00	1.00	1.00
Prokaryotic	Asgard archaeal	1.08	0.574	0.663
Prokaryotic	Archaeal (other)	2.16	3.48E-09	1.30E-08
Alphaproteobacterial	Asgard archaeal	1.07	0.739	0.792
Alphaproteobacterial	Archaeal (other)	2.15	0.00106	0.00199
Asgard archaeal	Archaeal (other)	2.01	1.00E-08	3.01E-08

Supplementary Table 10. Pairwise comparisons of functional categories of KOGs with shared introns regarding intron type.

Category 1	Category 2	Odds ratio	*P* value	Adjusted *P* value
Cellular processes and signalling	Information storage and processing	1.20	0.727	0.727
Cellular processes and signalling	Metabolism	8.89	0.00942	0.0282
Information storage and processing	Metabolism	7.41	0.0373	0.0560

Supplementary Table 11. Pairwise comparisons of functional categories of Pfam duplications with introns regarding intron type.

Category 1	Category 2	Odds ratio	*P* value	Adjusted *P* value
Cellular processes and signalling	Information storage and processing	2.19	0.0620	0.0930
Cellular processes and signalling	Metabolism	3.27	0.0170	0.0511
Information storage and processing	Metabolism	1.50	0.560	0.560

Supplementary Table 12. Comparisons of phylogenetic origins of Pfam duplications with an intron history regarding intron type.

Phylogenetic origin 1	Phylogenetic origin 2	Odds ratio	*P* value	Adjusted *P* value
Bacterial (other)	Asgard archaeal	2.09	0.0731	0.157
Bacterial (other)	Archaeal (other)	2.87	0.0420	0.105
Bacterial (other)	Eukaryotic	3.41	2.37E-10	3.55E-09
Bacterial (other)	Alphaproteobacterial	inf	0.235	0.441
Bacterial (other)	Prokaryotic	inf	6.05E-06	4.54E-05
Asgard archaeal	Archaeal (other)	1.37	0.757	0.963
Asgard archaeal	Eukaryotic	1.63	0.310	0.517
Asgard archaeal	Alphaproteobacterial	inf	0.589	0.884
Asgard archaeal	Prokaryotic	inf	0.0144	0.0721
Archaeal (other)	Eukaryotic	1.19	0.770	0.963
Archaeal (other)	Alphaproteobacterial	inf	1.00	1.00
Archaeal (other)	Prokaryotic	inf	0.0420	0.105
Eukaryotic	Alphaproteobacterial	inf	1.00	1.00
Eukaryotic	Prokaryotic	inf	0.0421	0.105
Alphaproteobacterial	Prokaryotic	nan	1.00	1.00

Supplementary References

1. Yenerall, P. & Zhou, L. Identifying the mechanisms of intron gain: progress and trends. *Biol. Direct* **7**, 1–10 (2012).
2. Hankeln, T., Friedl, H., Ebersberger, I., Martin, J. & Schmidt, E. R. A variable intron distribution in globin genes of *Chironomus*: evidence for recent intron gain. *Gene* **205**, 151–160 (1997).
3. Zhang, L.-Y., Yang, Y.-F. & Niu, D.-K. Evaluation of models of the mechanisms underlying intron loss and gain in *Aspergillus* fungi. *J. Mol. Evol.* **71**, 364–373 (2010).
4. Torriani, S. F. F., Stukenbrock, E. H., Brunner, P. C., McDonald, B. A. & Croll, D. Evidence for extensive recent intron transposition in closely related fungi. *Curr. Biol.* **21**, 2017–2022 (2011).
5. Burge, C. B., Padgett, R. A. & Sharp, P. A. Evolutionary fates and origins of U12-type introns. *Mol. Cell* **2**, 773–785 (1998).
6. Moyer, D. C., Larue, G. E., Hershberger, C. E., Roy, S. W. & Padgett, R. A. Comprehensive database and evolutionary dynamics of U12-type introns. *Nucleic Acids Res.* **48**, 7066–7078 (2020).
7. Basu, M. K., Makalowski, W., Rogozin, I. B. & Koonin, E. V. U12 intron positions are more strongly conserved between animals and plants than U2 intron positions. *Biol. Direct* **3**, 19 (2008).
8. Sharp, P. A. & Burge CB. Classification of introns: U2-type or U12-type. *Cell* **91**, 875–879 (1997).
9. Vosseberg, J. *et al.* Timing the origin of eukaryotic cellular complexity with ancient duplications. *Nat. Ecol. Evol.* **5**, 92–100 (2021).
