# Supplementary material for: The spread of the first introns in proto-eukaryotic paralogs
Source: Commun Biol. 2022 May 19;5:476. doi: 10.1038/s42003-022-03426-5 (PMC9120149; doi:10.1038/s42003-022-03426-5)
Supplement: Supplementary file 3 — Description of Additional Supplementary Files [file 42003_2022_3426_MOESM3_ESM.pdf]

Description of Additional Supplementary Files

File name: Supplementary Data 1
Description: KOG clusters

File name: Supplementary Data 2
Description: Species list with links to used genome annotation files

File name: Supplementary Data 3
Description: Pairwise comparisons of functions of pairs of KOGs with and without introns (unique versus shared introns).

File name: Supplementary Data 4
Description: Pairwise comparisons of functions of Pfam duplications with and without introns (duplications with versus without preduplication introns).

File name: Supplementary Data 5
Description: Pairwise comparisons of localisations of Pfam duplications with and without introns (duplications with versus without preduplication introns).

File name: Supplementary Data 6
Description: Pairwise comparisons of functions of pairs of Pfam OGs with and without introns (unique versus shared introns).

File name: Supplementary Data 7
Description: Pairwise comparisons of localisations of pairs of Pfam OGs with and without introns (unique versus shared introns).

File name: Supplementary Data 8
Description: Pairwise comparisons of functions of KOGs with shared introns regarding intron type.

File name: Supplementary Data 9
Description: Pairwise comparisons of functions of Pfam duplications with introns regarding intron type.

File name: Supplementary Data 10
Description: The source data behind the graphs in the paper
